# Individual differences in cognitive offloading: a comparison of intention offloading, pattern copy, and short-term memory capacity

**DOI:** 10.1186/s41235-021-00298-x

**Published:** 2021-04-29

**Authors:** Hauke S. Meyerhoff, Sandra Grinschgl, Frank Papenmeier, Sam J. Gilbert

**Affiliations:** 1 Leibniz-Institut Für Wissensmedien, Schleichstr. 6, 72076 Tübingen, Germany; 2 University of Tübingen, Tübingen, Germany; 3 University of Graz, Graz, Austria; 4 University College London, London, UK

**Keywords:** Individual differences, Reliability, Cognitive offloading, Intention offloading task, Pattern copy task

## Abstract

The cognitive load of many everyday life tasks exceeds known limitations of short-term memory. One strategy to compensate for information overload is cognitive offloading which refers to the externalization of cognitive processes such as reminder setting instead of memorizing. There appears to be remarkable variance in offloading behavior between participants which poses the question whether there is a common factor influencing offloading behavior across different tasks tackling short-term memory processes. To pursue this question, we studied individual differences in offloading behavior between two well-established offloading paradigms: the intention offloading task which tackles memory for intentions and the pattern copy task which tackles continuous short-term memory load. Our study also included an unrelated task measuring short-term memory capacity. Each participant completed all tasks twice on two consecutive days in order to obtain reliability scores. Despite high reliability scores, individual differences in offloading behavior were uncorrelated between the two offloading tasks. In both tasks, however, individual differences in offloading behavior were correlated with the individual differences in an unrelated short-term memory task. Our results therefore show that offloading behavior cannot simply be explained in terms of a single common factor driving offloading behavior across tasks. We discuss the implications of this finding for future research investigating the interrelations of offloading behavior across different tasks.

## Introduction

If you have a doctor’s appointment in two weeks, do you create a reminder in the calendar of your smartphone? Or, if you bake a cake, do you set an alarm to remind you that you should remove the cake from the oven before your smoke detector does so? If your answer to these (or similar) questions is “yes,” you are among the vast majority of people who use external aids to support memorization processes (Finley et al., 2018). In cognitive research, such behavior is referred to as cognitive offloading, namely “the use of physical action to alter the information processing requirements of a task so as to reduce cognitive demand” (Risko & Gilbert, 2016). Cognitive offloading is ubiquitous in many aspects of everyday life supporting a wide variety of cognitive processes including perception (e.g., Risko et al., 2014), memory (e.g., Gilbert, 2015a), problem solving (e.g., Moritz et al., 2020), mental arithmetic (e.g., Goldin-Meadow et al., 2001; Osiurak et al., 2018), navigation (e.g., Fenech et al., 2010), or spatial reasoning (e.g., Armitage et al., 2020; Chu & Kita, 2011; Weis & Wiese, 2018, 2019). Incorporating external aids into cognitive processing has been conceptualized as extended mind (Clark & Chalmers, 1998). For instance, by offloading memory processes, the offloading individual creates a human–technology transactive memory system in which information is distributed between internal and external memory resources (Wegner & Ward, 2013).

In this project, we focus on memory offloading which is probably the most common form of cognitive offloading. Memory offloading typically results in increasing accuracy or efficiency in solving the task at hand (i.e., “effects with technology”; Salomon, 1990). For instance, offloading allows for a more accurate solving of demanding short-term memory tasks (e.g., Risko & Dunn, 2015) as well as for more efficient solution of arithmetic problems (e.g., Cary & Carlson, 2001; Osiurak et al., 2018) or information extraction problems (Moritz et al., 2018). Applied to our opening example of cognitive offloading, noting your doctor’s appointment in your calendar would make it more likely that you actually show up for this appointment. However, research examining aftereffects of cognitive offloading (i.e., “effects of technology”; Salomon, 1990) has also identified potentially detrimental effects of cognitive offloading on the formation of memory representations (e.g., Eskritt & Ma, 2014; Grinschgl et al., in press; Henkel, 2014; Kelly & Risko, 2019a; Kelly & Risko, 2019b; Pyke & LeFevre, 2011; Sparrow et al., 2011) as well as the acquisition of problem solving skills (Moritz et al., 2020; O’Hara & Payne, 1998; van Nimwegen & van Oostendorp, 2009).

One of the most intriguing questions in research on cognitive offloading is why and when people offload information rather than memorize it. There appears to be consensus that both external factors such as demands of the task or characteristics of the external aid as well as internal factors such as metacognitive considerations alter the amount of offloading behavior (see Risko & Gilbert, 2016). Regarding external factors, an increasing amount of cognitive offloading has been observed with an increasing memory load (e.g., Arreola et al., 2019; Gilbert, 2015a; Risko & Dunn, 2015), increasing complexity and relevance (e.g., Schönpflug, 1986) as well as increasing difficulty (e.g., Hu et al., 2019). Further, reduced temporal cost of offloading (e.g., Fu & Gray, 2000; Gray et al., 2006; Patrick et al., 2015; Waldron et al., 2011) or more intuitive offloading tools (Grinschgl et al., 2020a) increase the proportion of offloaded information. Regarding internal factors, metacognitive considerations have been identified as being associated with offloading behavior (see Risko & Gilbert, 2016, for “a metacognitive model of cognitive offloading”). Broadly spoken, such metacognitive considerations reflect feelings and/or beliefs about one’s own internal ability to successfully solve a task without externalization. If such considerations result in a positive evaluation, this should lead toward a memory strategy (i.e., no offloading) whereas a negative evaluation should lead to an offloading strategy (see Arango-Muñoz, 2013). Recently, this view has received empirical support from research demonstrating shared variance between individual differences in metacognitive beliefs and offloading behavior (e.g., Boldt & Gilbert, 2019; Gilbert, 2015b; Hu et al., 2019; Risko & Dunn, 2015; Sachdeva & Gilbert, 2020). It seems noteworthy, however, that the relationship between metacognitive considerations about one’s own memory reliability and cognitive offloading so far mostly has been demonstrated on the correlational level only and that direct manipulations of metacognitive evaluations did not always induce the corresponding effects on offloading behavior (Engeler & Gilbert, 2020; Grinschgl et al., 2020b; but see also Gilbert et al., 2020). Nevertheless, the correlational evidence indicates that individual differences might provide important contributions to the explanation of cognitive offloading.

In the present work, we take a broader perspective on individual differences in offloading behavior. Although cognitive offloading is studied with different research paradigms, the results (including our own) are typically considered to generalize across paradigms (e.g., Risko & Gilbert, 2016). The main argument for this is that the different paradigms are susceptible to similar manipulations; however, this does not imply that the different paradigms actually do measure the same tendency to offload (in the sense of individual differences). We therefore investigate whether individual offloading behavior in one task is related to individual differences in offloading behavior in another task. In other words, we ask whether the tendency of offloading information reflects a general habit which consistently emerges across different tasks. We are not aware of any previous study which has investigated this research question. As this is the first attempt to relate offloading behavior between tasks, we studied individual differences in standard variants (i.e., closely matched to previous studies using the corresponding tasks) of two of the most common paradigms in memory offloading: the intention offloading task and the pattern copy task.

The intention offloading task (originally reported in Gilbert, 2015a) tackles memory for intentions (sometimes also referred to as prospective memory; Brandimonte et al., 1996; Kliegel et al., 2008). During this task, the participants continuously move a set of objects across the lower border of a surrounding square in a mandatory order. A subset of these objects, however, is associated with a particular intention which requires that these objects are moved across one of the other boarders. For a memory-based solution of the task, the participants have to remember the intentions associated with this subset of objects so that they can move them across the correct border of the square when it is their turn to be moved. Importantly, however, in the critical conditions, the participants do not fully need to rely on their memory to fulfill these delayed intentions. Instead, they are allowed to set external reminders by moving the corresponding objects close to the border across at which they need to be moved later in the sequence. Offloading behavior is indicated by the externalizing proportion which is the relative frequency of reminder setting (i.e., moving objects toward the corresponding border in order to fulfill the delayed intention). Participants are more inclined to externalize the delayed intentions when memory load increases as well as when they expect interruptions while performing the task (Gilbert, 2015a). Generally, the participants in this task are biased to rely more on externalizations than would be optimal, given their unaided memory performance (Gilbert et al., 2020). In other words, the participants typically prefer to use external reminders than load internal short-term memory while doing this task. Offering a monetary incentive is capable of reducing this bias but does not eliminate it completely (Sachdeva & Gilbert, 2020). This suggests that non-metacognitive factors, such as a preference to avoid cognitive effort, also play a role in offloading behavior.

In contrast to the intention offloading task, the pattern copy task (originally reported as Blocks World Task in Ballard et al., 1992) tackles continuous short-term memory load. In this task, memory offloading reduces the amount of information that needs to be handled simultaneously. The participants copy a pattern of colored squares from a model window to a workspace while only one of the windows is visible at the same time. Critically, the number of the to-be-copied squares clearly exceeds the documented capacity limitations of short-term memory (e.g., Cowan, 2001), so that the participants have to switch back and forth between the two windows. When performing the task, more pronounced offloading is indicated by an increasing number of openings of the model window. This fits the definition of cognitive offloading because increased physical action (switching between the windows more often) implies that a smaller amount of information needs to be stored in short-term memory during each copy cycle. Although the intention offloading task and the pattern copy task seemingly capture different aspects of short-term memory (i.e., remembering prospective intentions vs. loading short-term memory), there are some remarkable commonalities in the typically observed result patterns. As in the intention offloading task, the participants in the pattern copy task seem to be biased away from loading their short-term memory toward its limit. Instead, initial research on this task suggested that the participants tend toward a rather minimalistic memory strategy which is indicated by many openings of the model window (Ballard et al., 1992, 1995). However, when accessing the model window is associated with temporal costs, participants reduce their offloading behavior so that they appear to follow cost–benefit considerations rather than a minimal memory approach (Fu & Gray, 2000, see also Gray & Fu, 2004; Gray et al., 2006; Grinschgl et al., 2020a, 2020b; Patrick et al., 2015; Waldron et al., 2011). As the reduction in temporal costs also could be considered as an incentive, this reduction in offloading behavior in the pattern copy task also matches with the incentive-induced reduction in offloading behavior in the intention offloading task.

We chose to study the relations between individual differences in the intention offloading task and the pattern copy task as both tasks allow observers to offload short-term memory processes but focus on different aspects of memory. Whereas the intention offloading task focusses on a control mechanism that allow for switching between the ongoing task and fulfilling the delayed intentions, the pattern copy task focusses on storage capacity for feature-locations bindings. This distinction is made by most of the common models of working memory (e.g., Baddeley, 1992; Engle, 2002) as well as prospective memory (e.g., Smith & Bayen, 2004). We considered the interrelations of individual differences in these tasks to be the most informative for future research as the outcome is rather unclear. On the one hand, both tasks allow for offloading memory processes so that individual differences might be related. On the other hand, however, both tasks tap different aspects of memory so that it also is possible that individual differences are unrelated between the tasks. Thus, whether or not individual differences between those tasks are related will inform (and hopefully inspire) future research on individual differences in cognitive offloading as it indicates whether the tendency to offloading could potentially spread across heterogeneous paradigms or whether it is rather narrowly constrained.

Beyond the correlation of individual differences in offloading behavior between the intention offloading task and the pattern copy task, we also aimed at studying how these individual differences are related to individual differences in short-term memory capacity. Such a relation appears plausible as cognitive offloading could potentially compensate for a lower internal capacity of short-term memory. Indeed, Gilbert (2015a) who studied the intention offloading task as well as Risko and Dunn (2015) who studied a memory span test (in which participants were allowed to offload short-term memory by note taking) observed initial evidence for this proposed relationship. In both studies, unaided performance in a block in which offloading was not allowed was inversely correlated with the amount of offloading in a block in which participants were allowed to freely choose their offloading behavior. Beyond such correlations in the same task, however, we are aware of only one study which has approached this question with an independent estimate of working memory capacity. Morrison and Richmond (2020) repeated the study of Risko and Dunn (2015) and extended it by adding working memory estimates from two span tasks. Whereas they were able to replicate the experimental findings of Risko and Dunn (2015), they did not observe any correlation between memory capacity and offloading behavior; neither for the independent estimates of memory capacity, nor for unaided performance in the same task. The conflicting results such as this one clearly urge for further research. We therefore implemented a short-term memory test in our chain of tasks to further pursue this research question. We chose a variant of the Corsi blocks task as this task is (a) well established in research studying short-term memory storage (Della Sala et al., 1999) and (b) does not favor one of our offloading tasks in terms of overlapping features. (The spatial component might be a bit more related to the pattern copy task whereas the sequential component might be a bit more related to the intention offloading task.)

One central challenge in interpreting the previous results on correlations between offloading behavior and short-term memory capacity is the general lack of estimates of reliability. However, reliable measures are a necessary prerequisite for studying individual differences. In fact, all involved measures must capture individual differences reliably because reliability limits the potentially observable correlations with other measures (Nunnally, 1970; Spearman, 1904). As recently demonstrated by Hedge et al. (2018), reliable estimates of individual differences cannot be taken for granted in most paradigms emerging from experimental approaches as these paradigms typically are designed to minimize variance from individual differences in order to maximize the variance emerging from experimental manipulations. Correlational approaches (such as individual differences), however, require sufficient variance between participants so that these differences could emerge stably at different points in time. Although experimental and correlational approaches do not necessary exclude each other (e.g., Meyerhoff & Papenmeier, 2020), the reliability of measures emerging from experimental paradigms needs to be established empirically. To do so, our study was organized in two sessions. In both sessions, the participants completed all three tasks (with different sets of trials) so that the correlation of individual differences across both sessions provide a direct estimate of reliability.

## Method

We have preregistered this study at the Open Science Framework (https://osf.io/pbrqj). Raw data, scripts for data analysis, as well as scripts for all materials are available at https://osf.io/c7gfw/.

### Participants

The final sample consisted of 65 students of the University of Tübingen (51 female, 14 male, 19–31 years) who were recruited from our regular participant pool. The participants received course credit or monetary compensation for their participation in the two sessions (approx. 1 h each). The experimental procedure was approved by the institutional review board of the Leibniz-Institut für Wissensmedien. All participants signed informed consent prior to testing.

### Power considerations and deviations from the preregistration

With regard to statistical power, the most relevant statistical test in this project is the correlation between the individual differences in offloading behavior in the intention offloading task and the pattern copy task. We considered this correlation to be relevant when *r* > 0.35^1^ and aimed at detecting this correlation with a power of (1 − *β*) > 0.8 at *α* = 0.05 (two-tailed). These considerations result in a minimum sample size of 59 participants (G*Power, Faul et al., 2007). We therefore preregistered a sample size of 60 complete data sets. According with this preregistration, we aimed at replacing participants with incomplete data (2 participants), who failed to comply with the task instructions (e.g., no offloading at all in the intention offloading task; 3 participants), or who were insensitive to the incentive structure in the intention offloading task (3 participants). While attempting to replace these participants, the spread of the COVID-19 pandemic stopped any laboratory activity. At that point, we had collected data from a total of 65 participants, but two of them only completed the first session. As we were unable to finish the study as intended, we deviated from the preregistered inclusion criteria as follows.

In order to maintain the highest power of the study, we therefore did not exclude and replace the entire data from individual participants as preregistered but excluded only disputable data from individual tasks of these participants (please see results section for further details). With this new strategy, we preserved partial data from participants who had disputable data in only a subset of all tasks. By doing so, we were able to include 56 data sets in the critical correlation in the first session, and 53 data sets in the same correlations in the second session. Therefore, the a priori power remained at an acceptable level of (1 − β) > 0.78 in the first session and (1 − β) > 0.76 in the second session.

### Apparatus

The study was conducted on 12.3″ Microsoft Surface Pro Tablets (2736 × 1824 pixels). The tablets were lying flat on a table resulting in a viewing distance of approximately 36 cm. (All degrees of visual angle are calculated based on this distance.) The touch screen of the tablets served as input device. All tasks were coded using the PsychoPy 3.2.4 libraries (Peirce, 2007).

### Stimuli

#### Intention offloading task

**Fig. 1:**
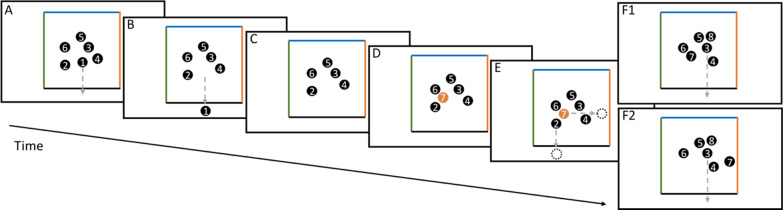
Illustration of the intention offloading task. A: A set of six circles appears onscreen. B: The participants drag the circles in the order indicated by the numbers. C: Dragged circles disappear from the display. D: For each removed circle, a new circle appears (until a total of 25 circles had been presented). New circles can be associated with an intention (indicated by a 2 s color cue). These circles need to be dragged across the correspondingly colored border of the frame when it is their turn. E: For circles which are associated with an intention, the participants can decide whether to rely on an internal memory strategy (see F1), or whether they externalize the intention by locating the circle near the corresponding border thus generating an external reminder (see F2). Cognitive offloading is indicated by a larger number of externalizations

This task served to measure offloading behavior for delayed intentions. In each trial, the participants dragged 25 circles (1.8 deg in diameter) across the border of a surrounding square frame (22 × 22 deg). The circles had to be dragged in the order indicated by numbers from 1–25 displayed on top of them. When the participants deviated from the correct order, the dragged circle jumped back to its previous location while turning red for 200 ms. Circles which were dragged in the correct order faded out within 300 ms. At the beginning of each trial, the first six circles appeared in a rapid sequence of 100 ms for each circle. Thereafter, a new circle appeared whenever another circle was dragged out of the frame (e.g., removing circle 6 triggered the appearance of circle 12). New circles always appeared in the central area of the square (9.5 × 9.5 deg). Invisible to the participants, this central area was divided into 25 squares, each of which served as starting point for one object in a randomized order. The trial ended when the participant had dragged the 25th circle after which no further circles appeared onscreen. The borders of the surrounding square were colored individually: the lower border was white, the left border was yellow, the upper border was pink, and the right border was blue. Out of the 25 circles, 15 were *standard circles* which needed to be dragged across the lower border of the surrounding square. The remaining objects were *delayed intention circles* which needed to be dragged across one of the other borders (left, upper, and right). When one of the delayed intention circles appeared onscreen, a surrounding color cue indicated the border across which this circle needed to be dragged when it was its turn (i.e., after removing the preceding circles). This cue was visible for 2 s. In order to fulfill the delayed intentions, the participants either could rely on a memory or an offloading strategy. With a memory strategy, the participants would leave the delayed intention circle in the central area of the screen and memorize the intention. With an offloading strategy, the participants would move the delayed intention circle closer to the corresponding border. The new spatial location of this circle then serves as an external reminder for fulfilling the intention. The task instructions explained both strategies explicitly. The participants could freely vary between these strategies across the different circles of each trial (see Fig. 1 for an illustration of the task).

Each participant completed 20 trials of the task preceded by one practice trial. We created two sets of trials in which we randomly assigned the delayed intentions to specific circles (one set for each session). We then repeated these sets of trials across all participants to avoid random variations in difficulty from overshadowing individual differences. In order to prevent participants from simply offloading all delayed intention circles, we introduced an incentive structure which we varied on a trial-by-trial basis. In each trial, the participants could reach a maximum of 115 points, one point for each correctly dragged standard circle, and up to 10 points for each correctly dragged delayed intention circle. For the delayed intention circles, the incentive varied between trials based on the chosen strategy. Correctly dragging a delayed intention circle was awarded with 1–10 points. The remaining points (i.e., 0–9) were awarded for not offloading a delayed intention circle before dragging it. For instance, in a trial in which correctly dragging a delayed intention circle was awarded with 4 points, 6 additional points could be earned by not offloading this circle (i.e., leaving it in the central area rather than moving it to the border of the surrounding frame). This resulted in 10 different incentive structures for delayed intention circles, each of which was repeated twice. A counter in the upper left corner of the screen indicated the number of earned points for the running trial. Beyond the increasing score, the participants received immediate feedback. When a circle was moved across one of the colored borders, the fading circle was filled with green color when it was dragged correctly and with red color when it was dragged incorrectly. The incentive structure of the upcoming trial was presented before the start of each trial. For the practice trial the incentive structure was 5 points for correctly dragged delayed intention circles plus additional 5 points for not offloading. We created two randomized orders of the incentive structures with the restriction that each incentive structure was used once in the first half of trials and once in the second half of the trials (one for each session). As with the trials, we repeated this order for all participants to prevent any effects of different orders from overshadowing individual differences.

As main dependent variable, we analyzed the proportion of externalizations of delayed intentions (i.e., offloading behavior) averaged across trials. For exploratory purposes, we also analyzed the accuracy in fulfilling the delayed intentions (i.e., performance).

#### Pattern copy task

This task served for measuring offloading behavior in a continuous short-term memory task. In each trial, the participants copied a pattern of colored squares from a model window on the left side of the screen to a workspace window on the right side of the screen. Both the model and the workspace window consisted of a 5 × 5 grid of individual squares with visible outlines (each 2.52° × 2.52°). Within the model window, twelve randomly selected squares were filled with the colors bisque, blue, cyan, dark green, green, gray, orange, pink, purple, red, sienna, and yellow (sampled without replacement). Whereas the workspace window was an empty grid at the beginning of a trial, an additional resource window below the workspace window consisted of twelve colored squares. The colors in the resource window (arranged in a 2 × 6 grid) matched the colors in the model window. In order to copy the pattern of colored squares displayed in the model window, the participants dragged-and-dropped the corresponding colored squares from the resource window into the workspace window.

**Fig. 2:**
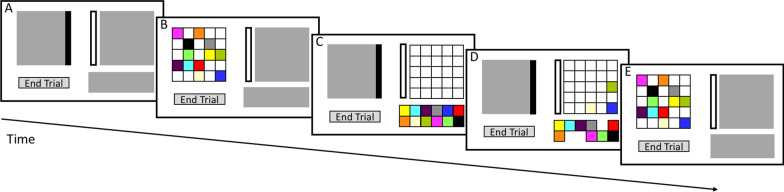
Illustration of the pattern copy task. The task of the participants is to copy the pattern from the model window (upper left) to the workspace window (upper right) by dragging-and-dropping the corresponding squares from the resource window (lower right). When participants move the black slider to open the model window, the workspace and resource windows are covered by gray masks (B & E). When the participants touch the white bar opening in order to uncover the workspace and resource window, the model window is masked (C & D). Cognitive offloading is indicated by an increasing number of openings of the model window

Importantly, the model window and the workspace/resource window were never visible at the same time. At the beginning of each trial, all windows were covered with gray masks. The participants could open the model window by moving a slider (a black bar) leftward across the model window and the workspace (and resource) window by clicking on a white bar next to it. Whenever the participants opened one of the windows, the other was immediately covered by the gray mask again; however, the participants were allowed to switch back and forth between the two views (model window and workspace/resource window) as often as they needed to. After copying the complete pattern, the participants pressed on a “End Trial”-button which was located below the model window. If the copied pattern was correct, the participants moved on to the next trial. Otherwise, they were asked to continue editing until the pattern was complete and correct (see Fig. 2 for in illustration of the task).

Each participant completed 20 trials of this task preceded by one practice trial. We created two sets of trials in which we randomly assigned the colored patterns (one set for each session). We then repeated the set of trials across all participants to prevent random variations in difficulty from overshadowing individual differences. The main dependent variable of this task is the number of openings of the model window with more openings indicating a higher amount of physical action, and a correspondingly reduced number of squares that need to be memorized at once. This variable therefore reflects a continuum from a more offloading-based strategy to a more memory-intense strategy (with a limit of copying all 12 squares at once). Secondary indicators of offloading behavior are the duration of the first opening of the model window (longer opening indicates less offloading) as well as the number of correctly copied items following the first opening (i.e., without any constrains from previous copy-cycles; more copied items indicate less offloading). Additionally, we performed an exploratory analysis of trial duration as a proxy for task performance.

#### Corsi blocks task

This task served for measuring short-term memory capacity operationalized as spatial span (adapted from Milner, 1971). In each trial, the participants memorized and rebuilt a sequence of spatial locations. These sequences were presented in a 5 × 5 grid of empty squares (each 2.52 × 2.52 deg) located in the center of the screen. During memorization, we presented a randomized sequence of the squares turned yellow for 1 s each. Following a retention interval of 300 ms, the participants rebuilt the sequence by tapping onto the corresponding squares in the same order. The length of the sequence adaptively varied with the performance of the participants. In the first trial, the length of the sequence was two squares. When the sequence was rebuilt correctly, the length of the sequence increased by one square, and it was shortened by one square when rebuilt incorrectly (with a minimum of two squares). Each participant completed 30 trials. As dependent variable, we analyzed the average length of the sequence in the last 10 correctly solved trials. We chose this average sequence length as dependent variable as it is less susceptible to random influences such as measurement errors than point-measures such as the maximum length. Further, averaging across multiple estimates results in a more precise measure as well as a continuous distribution of the estimates. Such a continuous distribution is more suitable for individual differences research rather than discrete estimates which only have a very restricted number of potential outcomes.

### Procedure

The experiment was divided into two identical sessions separated by 24 h. In both sessions the participants completed first the intention offloading task, then the Corsi blocks task, before ending with the pattern copy task. Presenting the tasks in a fixed order follows recent recommendations for best practice in individual differences research (in contrast to experimental research; see Goodhew & Edwards, 2019; Hedge et al., 2018) as altering task order might introduce (random) variance which might overshadow variance from individual differences thus reducing observable correlations. The participants were allowed to take brief breaks between the tasks. The task instructions were presented in written format (illustrated with schematic depictions), and an experimenter was present to resolve questions during instructions or practice.

## Results

### Data preparation

Table 1 lists the number of included participants for each session and task. We removed the data from participants who were classified as outliers (± 3 SD) either in the offloading and/or the performance measure of each individual task as these values would have a disproportionally large impact on the correlations (inflating reliability). Further, we probed whether the participants were sensitive to the incentive structure of the intention offloading task. We therefore calculated a correlation between the incentive for fulfilling a delayed intention (offloaded or not) and offloading behavior for the individual data of each participant. Please note that in trials with low incentives for correctly fulfilled intentions, the participants could earn more bonus points for not offloading the intention objects. Thus, a rational strategy is to offload more intentions in trials in which the majority of points stems from fulfilling the intentions independent of offloading behavior but to avoid offloading in trials in which the majority of points emerge from correctly fulfilled intentions which have not been offloaded before. We excluded the few participants for which the correlation coefficient was not numerically positive. (These participants offloaded randomly and thus were not following the task instructions; see Gilbert et al., 2020, for a similar exclusion criterion.) Finally, we had to exclude data which was not stored correctly. Descriptive statistics for all investigated variables are available in Table 2.

**Table 1:** Valid data sets and data exclusions separately for each task and session

	Valid samples	Outlier performance	Outlier offloading	Incentive structure	Missing data
*Session 1 (65 participants)*					
Intention offloading	59	1	4	1	–
Pattern copy task	62	1	2	–	–
Corsi blocks task	61	4	–	–	–
*Session 2 (63 participants)*					
Intention offloading	55	1	4	3	–
Pattern copy task	61	–	2	–	–
Corsi blocks task	58	3	–	–	2

**Table 2:** Descriptive statistics for all investigated variables

Task/measure	Mean (SD)		Range		Skewness		Kurtosis	
	Session 1	Session 2	Session 1	Session 2	Session 1	Session 2	Session 1	Session 2
*Intention offloading task*								
Externalizing proportion	.35 (.16)	.33 (.17)	.06–.69	.01–.68	0.39	0.16	2.19	2.07
Accuracy	.81 (.10)	.86 (.09)	.57–.97	.64–.98	− 0.47	− 0.76	2.31	2.98
*Pattern copy task*								
Openings model window	5.75 (1.61)	5.53 (1.68)	2.70–10.35	2.80–10.45	0.78	1.11	3.48	4.30
Duration first Opening	7.32 (4.05)	5.57 (2.77)	1.16–22.00	0.93–11.95	1.33	0.36	5.48	2.42
Copied during first opening	3.10 (0.82)	3.05 (0.76)	1.40–5.05	1.35–4.95	− 0.27	− 0.21	2.53	2.70
Trial duration	44.30 (10.88)	37.39 (7.64)	26.94–78.12	23.63–59.06	0.78	0.79	3.54	3.12
*Corsi blocks task*								
Capacity	4.59 (0.58)	4.78 (0.68)	3.3–6.4	3.3–6.9	0.49	− 0.03	3.89	3.74

### Reliability estimates

We calculated reliability scores for all relevant variables. As our major focus was on test–retest reliability, we report these analyses in full detail. However, further estimates of reliability are available in Table 3.

**Table 3:** Results of the reliability analysis

Task/measure	Test–retest [95% CI]	Cronbach’s α [95% CI]		Split-half [95% CI]	
		Session 1	Session 2	Session 1	Session 2
*Intention offloading task*					
Externalizing proportion	.70 [.54, .82]	.88 [.82, .91]	.91 [.87, .93]	.65 [.47, .78]	.77 [.63, .86]
Accuracy	.78 [.65, .87]	.88 [.84, .91]	.86 [.79, .89]	.71 [.55, .82]	.76 [.62, .86]
*Pattern copy task*					
Openings model window	.88 [.81, .93]	.95 [.94, .97]	.95 [.92, .97]	.86 [.78, .91]	.91 [.86, .95]
Duration first opening	.75 [.61, .84]	.95 [.93, .97]	.96 [.92, .98]	.74 [.60, .83]	.85 [.76, .90]
Copied during first opening	.82 [.73, .89]	.92 [.89, .94]	.93 [.89, .95]	.80 [.69, .87]	.88 [.81, .93]
Trial duration	.82 [.72, .89]	.95 [.93, .97]	.94 [.93, .96]	.84 [.74, .90]	.84 [.76, .90]
*Corsi blocks task*					
Capacity	.71 [.55, .82]	–	–	–	–

The Corsi blocks task was adaptive, so that responses within each session were not independent of each other. Thus, Cronbach’s α and split-half reliability were not calculated for this task

#### Intention offloading task

**Fig. 3:**
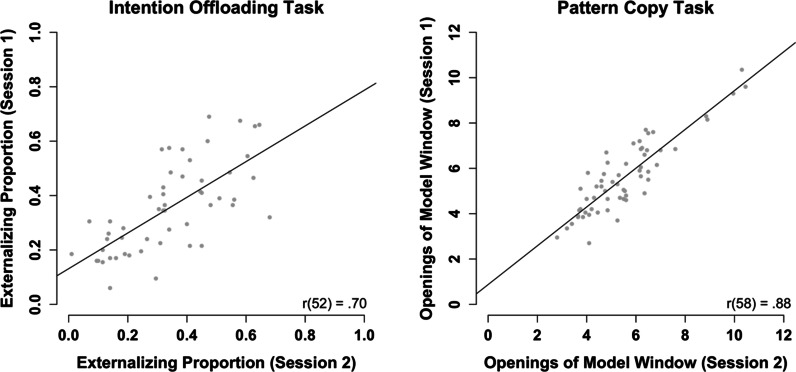
Reliability estimates of offloading behavior. The left panel displays the correlation of individual differences in the externalizing proportion in the intention offloading task. The right panel displays the correlation of individual differences in the number of openings of the model window in the pattern copy task

Within the intention offloading task, our primary focus was on offloading behavior as measured by the externalizing proportion (i.e., the proportion of offloaded delayed intentions). For this variable, we conducted the preregistered analysis of reliability by correlating individual externalizing proportions between both sessions (Pearson correlation; the combined data included valid data from 54 participants). As depicted in Fig. 3 (left panel), this analysis confirmed that the intention offloading task captures individual differences in the externalizing proportion reliably, *r*_P_(52) = 0.70, *p* < 0.001, 95% CI [0.54, 0.82].

Additionally, we conducted an exploratory analysis of task performance as measured by the accuracy of fulfilling delayed intentions. The individual differences in accuracy also emerged reliably, *r*_P_(52) = 0.78, *p* < 0.001, 95% CI [0.65, 0.87]. Finally, we probed the relationship between externalizing proportion and accuracy. Within the first session, we observed that a larger externalizing proportion was associated with more correctly fulfilled delayed intentions, *r*_P_(57) = 0.31, *p* = 0.016, 95% CI [0.06, 0.53], but we did not observe this correlation in the second session, *r*_P_(53) = 0.07, *p* = 0.624, 95% CI [− 0.20, 0.33].

#### Pattern copy task

Within the pattern copy task, our focus was on our primary variable for offloading behavior as measured by the number of openings of the model window. For this variable, we conducted the preregistered analysis of reliability by correlating individual differences in openings of the model window between both sessions (the combined data included valid data from 60 participants). As depicted in Fig. 3 (right panel), this analysis confirmed that the pattern copy task captured individual differences in the number of openings of the model window reliably, *r*_P_(58) = 0.88, *p* < 0.001, 95% CI [0.81, 0.93]. Individual differences in the secondary offloading variables duration of the first opening of the model window, *r*_P_(58) = 0.75, *p* < 0.001, 95% CI [0.61, 0.84], and the number of correctly copied items following the first opening, *r*_P_(58) = 0.82, *p* < 0.001, 95% CI [0.73, 0.89], also emerged reliably.

Additionally, we conducted an exploratory analysis of task performance as measured by the duration of trial completion. The individual differences in the duration of trial completion emerged reliably too, *r*_P_(58) = 0.82, *p* < 0.001, 95% CI [0.72, 0.89]. Finally, we probed the relationship between the number of openings of the model window and the duration of trial completion. Neither in the first session, *r*_P_(60) = − 0.08, *p* = 0.511, 95% CI [− 0.33, 0.17], nor in the second session, *r*_P_(59) = − 0.08, *p* = 0.540, 95% CI [− 0.33, 0.18], did we observe a correlation between these two dependent measures.

#### Corsi blocks task

For the Corsi blocks task, we conducted the preregistered reliability analysis. We correlated individual differences in the average length of the last 10 correctly solved sequences between both sessions (the combined data included valid data from 56 participants). This analysis confirmed that the Corsi blocks task captured individual differences reliably, *r*_P_(54) = 0.71, *p* < 0.001, 95% CI [0.55, 0.82].

### Correlations among offloading tasks

**Fig. 4:**
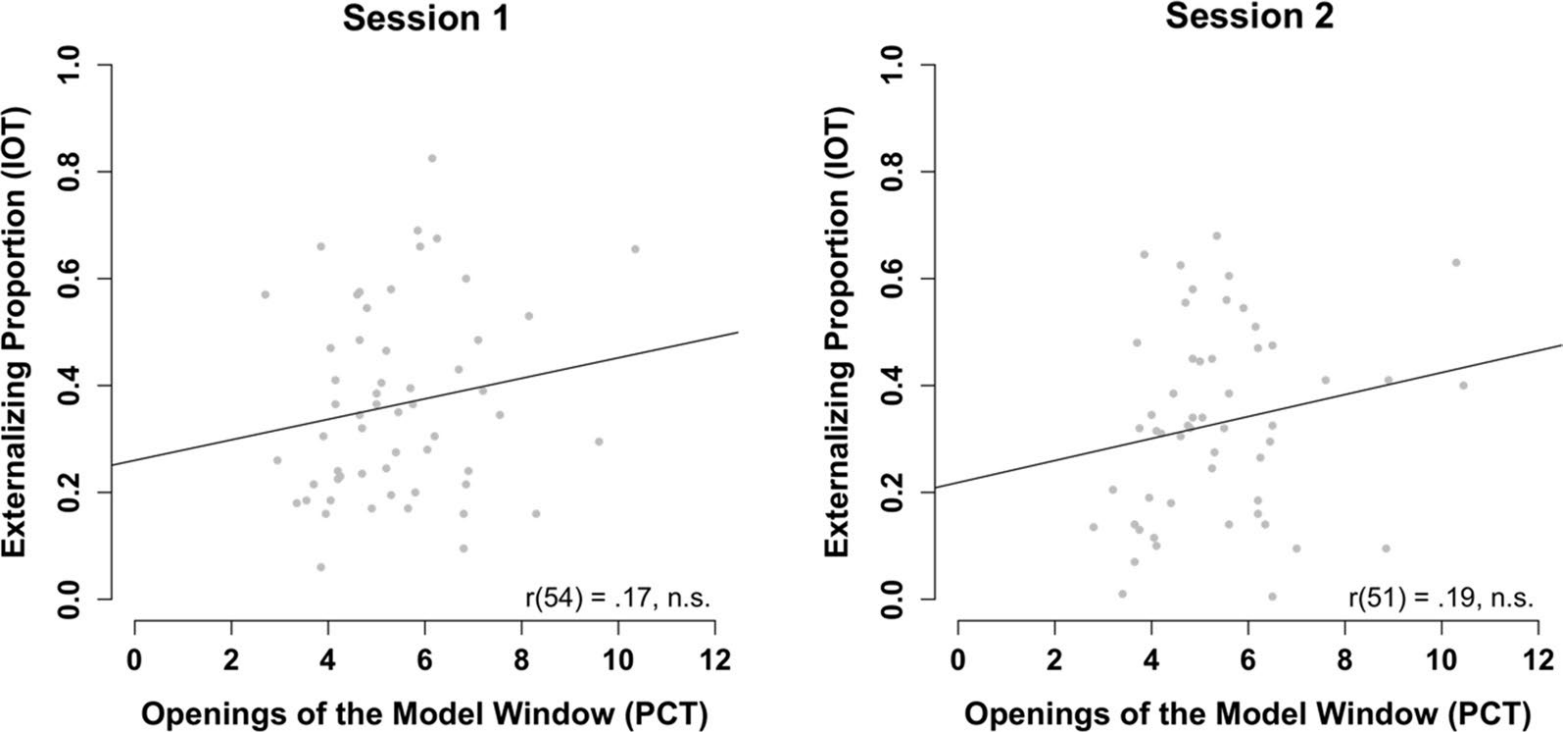
Depictions of the insignificant correlations between the cognitive offloading measures of the intention offloading task (IOT) and the pattern copy task (PCT)

Both paradigms, the intention offloading task and the pattern copy task measured individual differences in their conceptualizations of cognitive offloading reliably. Next, we conducted the preregistered analyses investigating whether offloading behavior in both offloading tasks is correlated. For these analyses, the combined pools of data included valid data from 56 participants in the first session and 53 participants in the second session. As depicted in Fig. 4, we did not observe significant correlations between the externalizing proportion in the intention offloading task and the number of openings of the model window in the pattern copy task in the first, *r*_P_(54) = 0.17, *p* = 0.216, 95% CI [− 0.10, 0.41], as well as the second session, *r*_P_(51) = 0.19, *p* = 0.175, 95% CI [− 0.09, 0.44]. There also were no correlations between the externalizing proportion of the intention offloading task and any of the secondary offloading variables of the pattern copy task (duration of the first opening of the model window; correctly copied items following the first opening), − 0.18 < all *r*_P_s < − 0.06, all *p*s > 0.199. Finally, an exploratory analysis of the performance data revealed that the individual differences in the proportion of correctly fulfilled delayed intentions in the intention offloading task were uncorrelated with the individual differences in the trial duration in the pattern copy task in the first, *r*_P_(54) = − 0.09, *p* = 0.499, 95% CI [− 0.35, 0.17], as well as the second session, *r*_P_(51) = − 0.05, *p* = 0.712, 95% CI [− .39, 0.15].

### Comparing correlations within and between tasks

The combined results of the reliability analysis and the comparison between tasks suggest that offloading behavior is correlated within the same task across the two sessions but not between tasks within the same session. In order to confirm this impression, we calculated Pearson and Filon’s z for the comparison of overlapping correlations based on dependent groups (Diedenhofen & Musch, 2015). For this comparison, we analyzed the data of the 51 participants for which we observed valid offloading data for both tasks in both sessions. The magnitude of the correlation of the externalizing proportion in the intention offloading task between the two sessions significantly exceeded the magnitudes of the correlations between the externalizing proportion (intention offloading task) and the number of openings of the model window (pattern copy task) in the first, *z* = 3.81, *p* < 0.001, as well as the second session, *z* = 3.12, *p* = 0.002. Similarly, the magnitude of the correlation of the number of openings of the model window in the pattern copy tasks between the two sessions significantly exceeded the magnitudes of the correlations between the externalizing proportion and the number of openings of the model window in the first, *z* = 5.31, *p* < 0.001, as well as the second session, *z* = 4.91, *p* < 0.001.

### Further exploration of the insignificant correlation between the offloading tasks

With our preregistered analysis, we observed no significant correlation between offloading behavior in both tasks in Session 1 as well as Session 2. Numerically, however, the corresponding correlation coefficients deviated from 0. Thus, the correlation might be too small to be observable within our study which is sufficiently powered only for correlations *r*_*P*_ > 0.35. In order to explore this possibility, we conducted four exploratory analyses that further evaluated the correlation between the externalizing proportion in the intention offloading task and the number of openings of the model window in the pattern copy task.

First, we calculated the Bayes factor (with a medium prior) for the correlations (Morey et al., 2018). For the first session, *r*_*P*_ = 0.17, *BF*_*10*_ = 0.61, as well as the second session *r*_*P*_ = 0.19, *BF*_*10*_ = 0.72, the Bayes factors are in the range of anecdotical evidence toward the null hypothesis. As this is in the range of inconclusive results, it does not rule out the possibility that offloading behavior between the tasks might be correlated at a numerically small level, however, as the Bayes factors are below 1, it also reveals no evidence in favor of such correlations.

Second, we pooled the data from both sessions, doubling the number of trials for each participant. For the pooled data, one would expect a substantial increase in the correlation between the offloading measures as the increase in the number of trials reduces measurement error and thus increases power and reliability. Pooling the data, however, only had a negligible effect on the correlation between offloading behavior in the intention offloading task and the pattern copy task, *r*_*P*_ (49) = 0.20, *p* = 0.162, *BF*_*10*_ = 0.76. This analysis therefore also does not provide evidence for the suggestion that we missed small correlations due to a lack of power.

Third, we explored the possibility that the small numerical correlations are residuals which might arise from the fact that offloading behavior in both tasks is related to short-term memory capacity. We therefore ran partial correlation analyses (Kim, 2015) which controlled for Corsi capacity. In these analyses, we observed reduced correlations between the offloading measures in both tasks for the first session, *r*_*P*_ (51) = 0.11, *p* = 0.424, the second session, *r*_*P*_ (48) = 0.15, *p* = 0.292, as well as the pooled data, *r*_*P*_ (45) = 0.16, *p* = 0.280. Given the very small numerical correlation values, these analyses again do not provide any evidence that we missed small correlations due to a lack of power.

Fourth and finally, because we could not take the reliability of our tasks into account when designing the study, we ran attenuation corrections in order to estimate the magnitude of the correlation coefficients that we would have observed if the tasks were perfectly reliable. Applying this correction (we used test–retest reliabilities as the most conservative estimate for reliability) showed that the correlation coefficients would increase from *r*_*P*_ = 0.17 to *r*_*P*_ = 0.22 in the first session, from *r*_*P*_ = 0.19 to *r*_*P*_ = 0.24 the second session, and from *r*_*P*_ = 0.20 to *r*_*P*_ = 0.25 with pooled data. The attenuation correction therefore shows that even if our tasks would have been perfectly reliable, the observed correlations still would have been substantially below the value of *r* = 0.35 for which we have powered our present study as we considered correlations of this magnitude to be relevant for the question whether offloading behavior reflects a stable tendency in individual difference across different tasks.

### Relationship between cognitive offloading and short-term memory

In order to explore the relationship between offloading behavior (in the intention offloading task as well as the pattern copy task) and short-term memory, we conducted the corresponding preregistered correlation analyses. Additionally, we performed an exploratory analysis of the relationship between task performance in both offloading tasks as well as short-term memory.

#### Intention offloading task

**Fig. 5:**
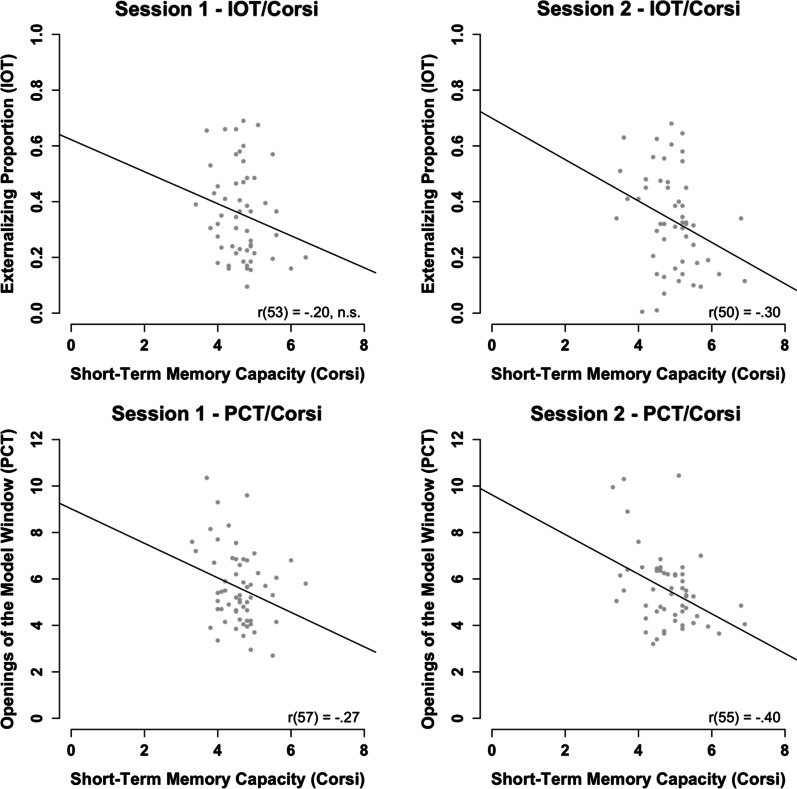
Correlations between offloading measures of the intention offloading task (IOT; upper panels) and the pattern copy task (PCT, lower panels) with short-term memory capacity measured by the Corsi blocks task separately for the first session (left panels) and the second session (right panels)

For the correlations between the intention offloading task and the Corsi blocks task, the combined pools of data included valid data from 55 participants in the first session and 52 participants in the second session. With regard to offloading behavior, the externalizing proportion in the intention offloading task was uncorrelated with the capacity in the Corsi blocks task in the first session, *r*_P_(53) = − .20, *p* = 0.145, 95% CI [− 0.44, 0.07]; however, in the second session this correlation was significant, *r*_P_(50) = − 0.30, *p* = 0.033, 95% CI [− 0.53, − 0.02]. Both correlations are depicted in Fig. 5 (upper panels). This finding suggests that participants with a higher short-term memory capacity rely on more memory-based strategies when addressing the delayed intentions (i.e., less offloading) than participants with a lower short-term memory capacity (at least in the second session). With regard to performance, the accuracy of correctly fulfilled delayed intentions was significantly correlated with the capacity in the Corsi blocks task in the first session, *r*_P_(53) = 0.27, *p* = 0.043, 95% CI [0.01, 0.50], as well as the second session, performance, *r*_P_(50) = 0.37, *p* = 0.008, 95% CI [0.10, 0.58]. This finding shows that participants with a higher short-term memory capacity perform more accurately in the intention offloading task than participants with a lower short-term memory capacity.

#### Pattern copy task

For the correlations between the pattern copy task and the Corsi blocks task, the combined pools of data included valid data from 59 participants in the first session and 57 participants in the second session. With regard to offloading behavior, the number of openings of the model window in the pattern copy task was negatively correlated with the capacity in the Corsi blocks task in the first session, *r*_P_(57) = − 0.27, *p* = 0.041, 95% CI [− 0.49, − 0.01], as well as in the second session, *r*_P_(55) = − 0.40, *p* = 0.002, 95% CI [− 0.59, − 0.15]. Both correlations are depicted in Fig. 5 (lower panels). This finding shows that participants with a higher short-term memory capacity rely on more memory-based strategies when copying the pattern (i.e., less offloading) than participants with a lower short-term memory capacity. With regard to performance, the negative correlation between task duration in the pattern copy task and capacity in the Corsi blocks task was significant in the first session, *r*_P_(57) = − 0.34, *p* = 0.007, 95% CI [− 0.55, − 0.09], as well as the second session, *r*_P_(55) = − 0.38, *p* = 0.007, 95% CI [− 0.58, − 0.13]. This finding shows that participants with a higher short-term memory capacity complete the pattern copy task faster than participants with a lower short-term memory capacity.

### Group-level analysis of offloading behavior and performance

A surprising finding in our data is that there was hardly any correlation between offloading behavior and performance on the individual differences level (we will return to this in the Discussion). In order to further explore the relationship between cognitive offloading and task performance, we therefore exploratorily analyzed our data on the group level for both tasks (i.e., averaged across participants).

#### Intention offloading task

**Fig. 6:**
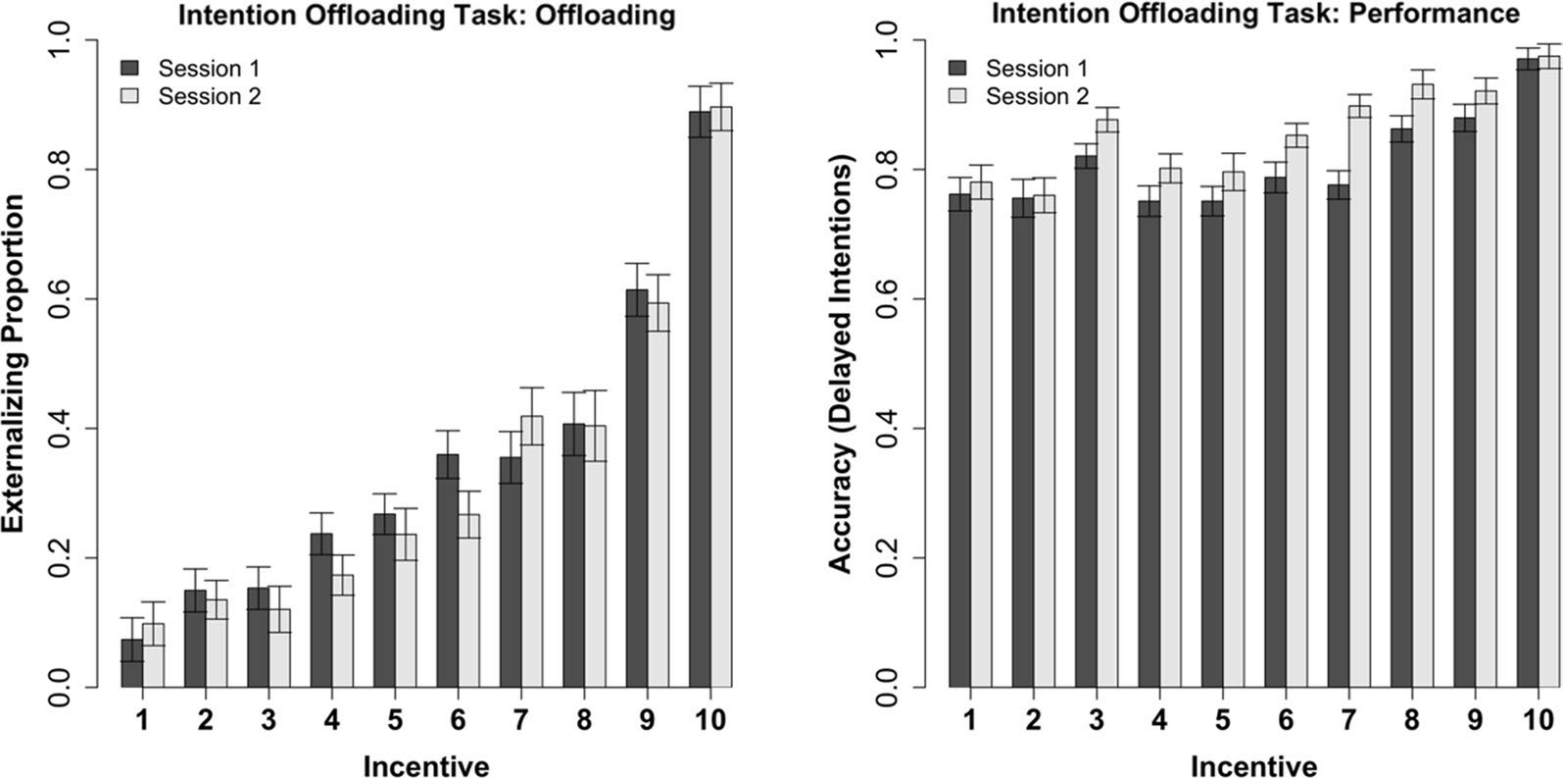
Group-level analysis of offloading behavior (externalizing proportion) and performance (accuracy of fulfilling delayed intentions) for the intention offloading task. Incentive refers to the number of points a participant received for correctly fulfilling a delayed intention object (offloaded or not). When the incentive was below 10, participants earned additional bonus points for using their memory instead of offloading

For the intention offloading task, we analyzed the impact of the incentive for correctly fulfilling delayed intentions on the externalizing proportion as well as accuracy (see Fig. 6) using linear mixed effect models (LME; Pinheiro et al., 2017). This analysis was inspired by previous work demonstrating that the incentive structure modulates offloading behavior in this task (e.g., Gilbert et al., 2020).

With regard to the externalizing proportion, this analysis revealed a significant slope for the incentive, *χ*^2^(1) = 1423.62, *p* < 0.001, demonstrating increasing offloading behavior with increasing incentive. There was no effect of the session, *χ*^2^(1) = 2.12 *p* = 0.145, nor an interaction between incentive and session, *χ*^2^(1) = 0.17, *p* = 0.677.

With regard to the accuracy of fulfilling delayed intentions, we also observed a significant slope for the incentive, *χ*^2^(1) = 268.80, *p* < 0.001, demonstrating increasing accuracy with an increasing incentive. Further, we observed more accurate performance in the second than the first session, *χ*^2^(1) = 46.53, *p* < 0.001, but no interaction between incentive and session, *χ*^2^(1) = 1.13, *p* = 0.287.

#### Pattern copy task

**Fig. 7:**
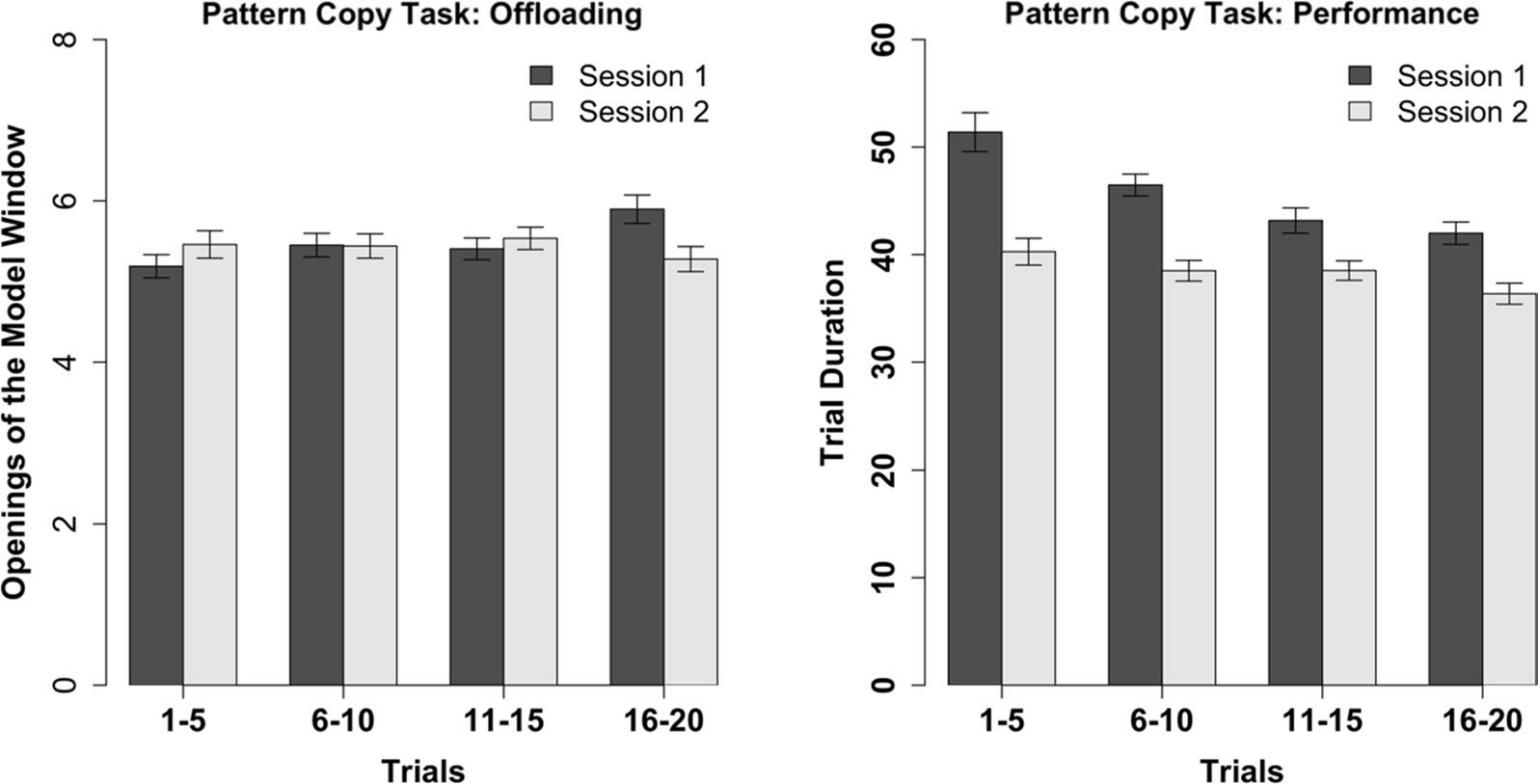
Group-level analysis of offloading behavior (openings of the model window) and performance (trial duration) for the pattern copy task. Please note that the statistical analyses used the trial number as a continuous variable

For the pattern copy task, we analyzed the development of the number of openings of the model window and trial duration across the time course of the experiment (again with an LME approach). This analysis was inspired by previous work indicating that experience with the pattern copy task alters offloading behavior (Grinschgl et al., in press). While the data are aggregated into bins covering quarters of the experiment for the visualizations in Fig. 7, we used the trial number as continuous variable for the statistical analyses.

With regard to the number of openings of the model window, we observed a significant slope of the trial number, *χ*^2^(1) = 9.49, *p* < 0.001, suggesting increasing offloading behavior over time. There was no effect of the session, *χ*^2^(1) = 0.74, *p* = 0.390, but we observed a significant interaction between the trial number and the session, *χ*^2^(1) = 19.43, *p* < 0.001. In order to further explore this interaction, we analyzed the slope of the trial number separately for the two sessions. This analysis showed that the slope of the trial number emerged in the first session, *χ*^2^(1) = 30.65, *p* < 0.001, but not in the second session, *χ*^2^(1) = 0.94, *p* = 0.333.

With regard to the trial duration, the analysis revealed a significant slope of the trial number, *χ*^2^(1) = 120.70, *p* < 0.001, signaling faster trial completions over time. Further, we observed an effect of the session, *χ*^2^(1) = 263.50, *p* < 0.001, signaling faster trial completions in the second than the first session, as well as an interaction between the trial number and the session, *χ*^2^(1) = 23.91, *p* < 0.001. In order to further explore this interaction, we analyzed the slope of the trial number separately for the two sessions. This analysis revealed that the slope of the trial number emerged in both sessions, but was more pronounced in the first session, *χ*^2^(1) = 106.31, *p* < 0.001, than in the second session, 27.66, *p* < 0.001.

## Discussion

We set out the current study to investigate the interrelations between individual differences in offloading behavior across different paradigms as well as their relations to individual differences in short-term memory capacity. The first major result of our study encompasses the suitability of the investigated tasks for individual differences research. Encouragingly, all evaluated measures provided good or even excellent reliability scores. This is true for the proxies of offloading behavior in the intention offloading task and the pattern copy task as well as for all proxies of task performance in the intention offloading task, the pattern copy task, and the Corsi blocks task. This is important as good reliability scores are necessary to interpret subsequent correlations (or their absence).

The second major result of our study is that individual differences in offloading behavior were not significantly correlated between the intention offloading task and the pattern copy task despite sufficiently reliable measures in both tasks. Numerically, the correlation coefficients were very small and did not increase substantially after pooling the data across both sessions as well as after an attenuation correction. As our study was not intended to capture correlations of such a small magnitude, it needs to be a task for future research to probe whether a potential correlation among offloading behavior might be very small, but present. Nevertheless, as both of our tasks were short-term memory tasks (although they capture different aspects of short-term memory), the insignificant correlations in our study make it unlikely that the tendency toward cognitive offloading can be explained in terms of a single factor that has a consistent influence on a wide variety of tasks covering perception (e.g., Risko et al., 2014), memory such as studied in this work, problem solving (e.g., Cary & Carlson, 2001; Moritz et al., 2018; Weis & Wiese, 2019), or even learning (Moritz et al., 2020; Storm & Stone, 2015).

A first potential objection against this interpretation might be that both tasks vary in several surface features (e.g., number of objects, the presence of explicit incentives, the presence of offloading cues), which might have reduced potential correlations. It seems unlikely, however, that these differences solely can explain our results as offloading behavior in both tasks correlated with short-term memory capacity as measured by the Corsi Block task which differs from both offloading tasks in even more surface features. As our observation is restricted to the comparison of two tasks only, it of course does not allow strong conclusions regarding the potentially multifaced underlying structure of cognitive offloading. Nevertheless, we would like to outline two alternatives of which we think they should receive attention from future research. The first alternative is that offloading behavior is consistent across different memory tasks only when they tap the same aspects of memory such as rather pure storage capacity or control/monitoring instances allocating limited resources (Baddeley, 1992; Engle, 2002; Smith & Bayen, 2004). Apparently, pursuing this alternative requires a systematic development of further offloading tasks as well as analyses which allow for identifying latent variables such as factor analysis or structural equation modeling. While this question exceeds the boundaries of our present project, our results can be informative for such a complex research project as it constrains the range of memory tasks which might load on particular latent variables. That is, we would predict that only tasks focusing on one particular aspect of short-term memory (or working memory) would share variance between observers. With regard to intention offloading, such tasks would also need to address the monitoring component of memory which allows to switch between fulfilling delayed intentions and an ongoing task (we are aware of one yet unpublished research project exploring multiple variations of the intention offloading task; see Ball et al., 2021). In contrast, with regard to the pattern copy task, such variations would need to focus on memory capacity, but might differ in surface features such as color and/or shape which typically show comparable effects in experimental studies on working memory (e.g., Meyerhoff et al., 2021).

The second alternative is that consistent offloading behavior is not bound to particular aspects of short-term memory but rather to the impact of metacognitive considerations on a particular task. In principle, such metacognitive considerations regarding one’s own (unaided) memory performance are highly erroneous. For instance, Beaudoin and Desrichard (2011) observed no correlation between self-estimated memory performance and actual memory performance in a meta-analysis. Metacognitions, however, may be of different relevance for different types of offloading tasks. On the one hand, metacognitive confidence in one’s own memory performance is inversely correlated with offloading behavior in the intention offloading task (Boldt & Gilbert, 2019; Gilbert, 2015b; but see Engeler & Gilbert, 2020). On the other hand, however, this relationship is more complicated in the pattern copy task. For this task, a recent study showed that reducing the metacognitive confidence with fake performance feedback increases the subjective impression of offloading behavior (i.e., the participants think they offloaded more) but leaves objectively measured offloading behavior unaffected (Grinschgl et al., , 2020b). It therefore remains possible that there is one group of offloading tasks in which metacognitive considerations affect offloading behavior and another group of offloading tasks in which metacognitive considerations affect only the subjective impression of offloading but leave objective measures of offloading behavior unaffected. Exploring this alternative would require the development of additional offloading tasks including an exploration of their relationship to metacognitive considerations about one’s own memory performance.

The third major result of our study addresses the correlation between offloading behavior and short-term memory capacity as measured with the Corsi blocks task. For both tasks, we observed inverse correlations between offloading behavior and short-term memory capacity (although the correlation was insignificant in the first session of the intention offloading task). This consistency suggests that offloading behavior is not detached from objective memory capacity but that individuals with lower memory capacity tend to compensate their lower internal capacity with an increasing amount of offloading.

Numerically, the inverse correlations between short-term memory capacity and offloading behavior were less pronounced for the intention offloading task. We think that this observation should be further explored in future research. On the one hand, such variations in the magnitude of the correlations could stem from peculiarities of the capacity estimate such as a closer link between the Corsi span capacity and the capacity-based pattern copy task. In this case one might observe the reversed pattern when the Corsi blocks task would be replaced by more complex span tasks which involve components of task switching (see Engle, 2002). On the other hand, a stronger impact of metacognitions on the intention offloading task relative to the pattern copy task might reduce the correlation with (objective) memory capacity. Irrespective of potential differences between the tasks involved in our study, however, our results contrast with those of Morrison and Richmond (2020) who observed no correlation between offloading behavior in a memory span test and memory capacity. As the investigated paradigms differ remarkably between both studies, we can only speculate about the cause of the deviating result patterns. Among the potential reasons are a lack of reliability of offloading behavior in the memory span test (which would diminish any correlation) as well as the possibility that not all offloading behavior in all tasks is linked to memory capacity.

One further observation from the correlations between individual differences in offloading behavior and short-term memory capacity in our study is that these correlations were numerically more pronounced in the second session than in the first session within both offloading paradigms. As our study was not intended to study such changes over time, we do not want to overemphasize this exploratory observation; however, we believe that it warrants follow-up research. The interesting question to study here is whether the association between offloading behavior in a particular task and short-term memory capacity strengthens over time because increasing experience with a task brings metacognitive considerations closer to actual memory performance.

Although the major focus of our study was on offloading behavior, we also collected performance data which we have considered in exploratory analyses. These analyses mirrored those of the offloading behavior. Individual differences in performance in the intention offloading task as well as in the pattern copy task emerged reliably but were uncorrelated between the tasks. The individual differences in performance, however, were correlated with the individual differences in short-term memory capacity for both tasks. Probably the most remarkable finding involving task performance is that offloading behavior and actual performance were mostly independent of each other. (There only was a correlation in the first session of the intention offloading task.) This observation is consistent with a matching result in Morrison and Richmond (2020). The most intriguing interpretation for this observation is that in particular those participants who would perform rather poorly without offloading tend to display more offloading behavior which in return might wash out the correlation of individual differences between offloading behavior and actual performance (Gilbert, 2015b). From this point of view, the presence of a correlation between the externalizing proportion and intention fulfillment accuracy in the first session, but its absence in the second session of the intention offloading task suggests that some practice with the task is necessary in order to wash out the correlation.

In order to further support the interpretation that particularly participants who perform poorly under unaided conditions benefit from offloading, we have analyzed the offloading and performance measures of our tasks on the group level (i.e., averaged across participants). In line with the vast majority of research on cognitive offloading (see Risko & Gilbert, 2016), this analysis showed that offloading behavior in general improved performance (i.e., on average the participants performed faster/more accurate in those conditions in which they also relied on offloading more intensively). Combining this observation with the increased offloading behavior of participants with lower short-term memory capacity and the mostly absent correlations between offloading behavior and performance in the individual difference data therefore suggests that indeed participants who would perform poorly under unaided conditions benefit from cognitive offloading.

## Conclusion

We explored the interrelations between individual differences in two common paradigms investigating cognitive offloading and observed rather unambiguous results. While both the intention offloading task as well as the pattern copy task revealed good-to-excellent reliability to study individual differences in offloading behavior, these individual differences were uncorrelated between the tasks. Individual differences in offloading behavior, however, were correlated with short-term memory capacity in both tasks. These results show that offloading behavior cannot simply be explained in terms of one common factor influencing different tasks (even when both address short-term memory processes). One practical implication of this is that interventions that alter individuals’ use of cognitive tools in one task cannot be assumed to generalize to another one, suggesting the importance of task-specific interventions. Future research is necessary to reveal factors which might contribute to the consistency and divergence of offloading behavior across different tasks.

## Data Availability

Raw data, scripts for data analysis, as well as scripts for all materials are available at https://osf.io/c7gfw/.

## Footnotes

1 Please note that this correlation refers to the actually observable correlation. As tasks typically are not perfectly reliable, the true correlation might need to be higher (by a value that was unknown prior to the study).
